# Achene heteromorphism in *Bidens pilosa* (Asteraceae): differences in germination and possible adaptive significance

**DOI:** 10.1093/aobpla/plz026

**Published:** 2019-06-03

**Authors:** Keliang Zhang, Linjun Yao, Yin Zhang, Jun Tao

**Affiliations:** Jiangsu Key Laboratory of Crop Genetics and Physiology, College of Horticulture and Plant Protection, Yangzhou University, Yangzhou, PR China

**Keywords:** *Bidens pilosa*, non-deep physiological dormancy, seed dimorphism, seed germination, weeds

## Abstract

*Bidens pilosa* (Asteraceae), a noxious weed in many ecosystems worldwide, produces large amounts of heteromorphic (central and peripheral) achenes. The primary aims of the present study were to compare the morphological, dormancy/germination characteristics of dimorphic achenes. Temperatures simulating those in the natural habitat of *B. pilosa* were used to test for primary dormancy and germination behaviour of fresh central and peripheral achenes. The effects of cold stratification, gibberellic acid (GA_3_) and dry storage on breaking dormancy were tested and the germination percentage of dimorphic achenes in response to osmotic stress was measured. Cold stratification, GA_3_ and dry storage significantly increased the germination percentage, suggesting both types of achenes had non-deep physiological dormancy. Variously pretreated central achenes had significantly higher germination percentages than peripheral achenes. Central achenes were more osmotically tolerant than peripheral achenes with a high germination percentage in high polyethylene glycol concentrations. These above differences among dimorphic achenes of *B. pilosa* increased the species’ fitness to adapt to heterogeneous habitats creating an ecological adaptive strategy that may allow *B. pilosa* to successfully thrive in stressful habitats.

## Teaser

*Bidens pilosa* (Asteraceae), a noxious weed in many ecosystems worldwide, produces large amounts of heteromorphic (central and peripheral) achenes. The present study demonstrated that the two distinct types of achenes differ in morphology, germination behaviour, dormancy and osmotic tolerance. The central achenes are dispersed for longer distances and have relatively shallow seed dormancy, whereas peripheral achenes have low dispersal ability and relatively strong seed dormancy. These differences represent the combination of different adaptive strategies in one plant and may have ecological significance for its successful survival.

## Introduction

Environmental heterogeneity creates a natural selection pressure that increases the complexity of plant growth and reproduction (Gutterman 2002). Plants interacts with cues from the external environment during long-term evolution and development and have developed unique seed dispersal and dormancy/germination strategies that allow them to adapt to unpredictable environmental conditions; these special mechanisms are often important aspects of seedling survival and development of these species (Gutterman 2002; Baskin and Baskin 2014). The production of heteromorphic diaspores in a plant species may result from a combination of strategies related to dispersal and germination; sometimes, these represent unique combinations of opposing strategies (Venable 1985; Imbert 2002).

Diaspore heteromorphism, a condition where a genotype produces different morphophysiological types of seeds, has been interpreted as an adaptive trait in heterogeneous environments (Venable 1985; Imbert 2002; Baskin and Baskin 2014). Not only do the diaspores of heteromorphic species look different, but they may differ in numerous other ways, including size/mass (Ellner and Shmida 1984; Venable and Levin 1985; Rocha 1996; Liyanage *et al.* 2016); dispersal ability (Venable 1985; Rocha 1996; Mandák and Pyšek 2001; Zhang *et al.* 2015); dormancy and germination (Corkidi *et al.* 1991; Wang *et al.* 2008; Baskin and Baskin 2014; Zhang *et al.* 2015; Liu *et al.* 2018); and the reproductive allocation of plants grown from heteromorphic seeds (Cheplick and Quinn 1988; Liyanage *et al.* 2016; Zhang *et al.* 2017). These differences range from autecological and population traits to genetic and molecular aspects of biology (Baskin and Baskin 2014).

The existence of heteromorphic seeds has been well documented among weeds, especially in the Amaranthaceae (including Chenopodiaceae) and Asteraceae (Imbert 2002; Wang *et al.* 2008). Baskin and Baskin (2014) found the Asteraceae include many ‘more diaspore heteromorphic species than any other family’ and noted that ‘of the 218 heteromorphic species listed by Imbert (2002), 63.6 % of the species and 52.5 % of the genera belong to the Asteraceae, and most of them produce dimorphic achenes’.

*Bidens pilosa* (Asteraceae), a common annual herb, has stems 30–180 cm tall with flowers and fruit from July to October (Shi *et al.* 2011). The species originally occurred in temperate and tropical America and has been expanding it range across Europe since the 19th century. It is currently considered as a noxious weed in many ecosystems worldwide (Holm *et al.* 1977) growing on roadsides and in fields and villages at elevations below 2500 m (Shi *et al.* 2011). In China, this species occurs from Liaoning province in northeast China to Yunnan province in southwest China (28°42′51″N, 98°93′18″E to 41°06′81″N, 122°95′46″E) (Shi *et al.* 2011). This species produces two types of achenes within the same infructescence that differ in morphology (Shi *et al.* 2011). In the field, we have observed that peripheral achenes remain attached to the receptacle longer than the central ones. Therefore, it is reasonable to suspect that the ecophysiology in two types of achenes also could be different.

Forsyth and Brown (1982) have shown that the dimorphic achenes of *B. pilosa* exhibited different germination characteristics. The long (central) achenes were found to germinate readily while the short (peripheral) achenes showed fairly exacting germination requirements. However, the achenes they used were not freshly matured but stored in the dark for 14 days post-harvest prior to germination (Forsyth and Brown 1982). If seeds have non-deep physiological dormancy (PD), after-ripening can occur during dry storage, especially at room temperatures (Baskin *et al.* 2013). Moreover, little is known about the class, level and type of dormancy in this species while the germination strategy of those dimorphic achenes remains unclear.

Annual plants survive by mechanisms used to ensure the appropriate timing of germination and germination occurs in a suitable habitat that will allow for seedling establishment (Gutterman 2002). For *B. pilosa*, the achene germination strategy may be the most significant factor determining survival because this is the only way their populations can be maintained. Understanding the germination behaviour of both types of achenes is likely to provide useful information related to the management of this weed. The main objective of the present study was to determine the dormancy and germination characteristics of the freshly matured dimorphic achenes. We hypothesized that *B. pilosa* has also developed a special strategy in the achene stage that is part of its suite of adaptations to unpredictable environmental conditions and achene dimorphism in *B. pilosa* aids the distribution of this species in time and space. To test this hypothesis, we compared (i) dormancy breaking and germination requirements of the freshly matured dimorphic achenes of this species and (ii) the ability of the two achene morphs to recover from drought stress.

## Materials and Methods

### Achene collection and field site description

Our study site is a woodland-open area located at the foot of Laoshan Mountain, Qingdao, China (36°16′95.37″N, 120°37′46.29″E; 19 m a.s.l.) that has been disturbed by human activity, in particular trampling. From 25 to 30 August 2017, freshly matured central and peripheral achenes of *B. pilosa* were collected from at least 100 individuals. In order to keep the experiments manageable, we restricted our experiments to the two ‘most extreme’ achene morphs: the outermost peripheral achenes vs. the most central ones, respectively. Achene germination experiments were started within 2 weeks after achene collection. Achene germination testing started immediately after harvest because achene germination behaviour may change during storage (Baskin and Baskin 2014).

The study area has a warm temperate monsoon climate, with a hot and rainy summer and a cold and dry winter. The average annual temperature is 12.7 °C, with January as the coldest month (−0.5 °C) and August the hottest (25.3 °C). Extreme maximum and minimum temperatures are 38.9 and −16.9 °C, respectively. Average annual rainfall is about 662 mm, 57 % of which is concentrated in summer (Wang 2005). On 26 August 2017, we used 10 1 × 1 m quadrats to survey the species associates of *B. pilosa* in the study area. The species associates of *B. pilosa* at the study site include the trees *Quercus acutissima* and *Robinia pseudoacacia*; the shrubs *Indigofera kirilowii*, *Lespedeza bicolor*, *Stephanandra incisa* and *Vitex negundo* var. *heterophylla*; and the herbs *Agrimonia pilosa*, *Artemisia japonica*, *Deyeuxia pyramidalis*, *Paraixeris denticulate*, *Sanguisorba officinalis* and *Thalictrum aquilegiifolium* var. *sibiricum*.

### Achene ratio and size

Twenty individual plants were chosen randomly in the natural habitat of *B. pilosa*; type and numbers of achenes were determined. Twenty groups each of 1000 freshly matured dimorphic achenes were weighed on an analytical balance (0.0001 g) (Sartorius BP 221 S, Sartorius, Germany) to determine achene mass. Achene length, width and height were measured with a vernier calliper.

### Imbibition of water by dimorphic achenes

Uptake of water under ambient laboratory conditions (20–25 °C, RH 40–55 %) was conducted to determine if there was difference between dimorphic achenes. Four replicates of 25 freshly matured dry achenes of each morph were weighed using analytical balance, and then the achenes were placed on two layers of Whatman No. 1 filter paper moistened with distilled water in 5-cm-diameter plastic Petri dishes. After 0.5, 1.0, 1.5, 2, 3, 4 and 5 h of water absorption, achenes were removed from the Petri dishes, blotted dry, reweighed and then put back into the Petri dishes. The percent increase in fresh mass (% *W*_*r*_) was calculated as % *W*_*r*_ = [(*W*_*f*_ − *W*_*i*_)/*W*_*i*_] × 100, where *W*_*i*_ is the initial achene mass and *W*_*f*_ the weight after a given time (Zhang *et al.* 2015).

### Effects of light and temperature on germination of dimorphic achenes

Four replicates of 25 freshly matured dimorphic achenes of each morph were incubated on two layers of Whatman No. 1 filter paper wetted with 3 mL distilled water in 5-cm-diameter plastic Petri dishes at 5/15, 10/20, 15/25 and 20/30 °C, respectively (12-h daily photoperiod) or continuous dark (achenes in black opaque bags). For achenes incubated under light, the higher temperature of alternating daily temperature coincided with the 12-h light period and the lower temperature with the 12-h dark period. These thermoperiods represent the mean daily maximum and minimum monthly temperatures: 5/15 °C, April; 10/20 °C, May and October; 15/25 °C, June and September; and 20/30 °C, July and August. Germination in light was checked daily for 30 days; germinated achenes were removed at each counting. Achenes incubated in dark were checked only after 30 days. After the germination, tetrazolium chloride was used to test the viability of non-germinated achenes and only viable achenes were used to calculate the germination percentage.

### Effect of cold stratification on germination of dimorphic achenes

Dimorphic achenes were arranged evenly on two layers of filter paper over ~5 cm of washed wet quartz sand (10–14 % moisture content) in a 10 cm deep × 20 cm diameter metal box. The box was closed and placed in refrigerator at 4 °C for 4, 8 and 12 weeks, after cold stratification; four replications of 25 dimorphic achenes each were incubated in light at 15/25 °C for 30 days. Germination was checked and germinated achenes were removed at each counting.

### Effect of dry storage (after-ripening) on germination of dimorphic achenes

To determine if dormancy is broken during after-ripening, dimorphic achenes were dry-stored for 0 (freshly matured achenes), 2, 4 and 8 weeks at room temperature (20–25 °C, RH 40–50 %) and tested for germination in light at temperature regime of 15/25 °C. Four replications of 25 freshly matured dimorphic achenes each were incubated in light at 15/25 °C for 30 days. Germination conditions were the same as those in the previous two experiments.

### Effect of GA_3_ on germination of dimorphic achenes

To test the effect of gibberellic acid (GA_3_) on dormancy breaking, four replications of 25 freshly matured dimorphic achenes each were incubated in 0 (distilled water), 5, 10 mg mL^−1^ GA_3_ solutions at 15/25 °C in light for 30 days. Germination conditions were the same as those in the previous two experiments.

### Effect of osmotic potential on germination of dimorphic achenes

To determine the effect of drought stress on the germination of dimorphic achenes, polyethylene glycol (PEG) was used to generate osmotic stress. Polyethylene glycol 6000 solutions were used in osmotic potentials of 0 (distilled water as control), −0.5, −1.0, −1.5, −2.0, −2.5, −3.0 MPa. Four replications of 25 freshly matured dimorphic achenes were incubated in each water potential. An amount of 3 mL PEG solution or distilled water were added to each 5-cm-diameter plastic Petri dishes with two layers of Whatman No. 1 filter paper; next, they were sealed with Parafilm to minimize evaporation of water from the solutions and incubated at 25 °C in light for 30 days.

### Statistical analysis

All analyses were performed with SPSS Version 18.0 (SPSS Inc., Chicago, IL, USA). Percentage data were arcsine-transformed before statistical analysis to ensure homogeneity of variance (non-transformed data are shown in figures). Other data were log_10_ transformed when necessary to improve normality and homogeneity of variances. Paired sample *t*-tests were used to compare the increase in achene mass after different times of imbibition and to analyse achene morphological traits and the effect of scarification on germination. One-way and two-way analysis of variance (ANOVA) was used to analyse other germination data. If ANOVA indicated significant differences in the data, Tukey’s HSD test was used to determine the differences between treatments (*P* < 0.05).

## Results

### Achene ratio and size

Each *B. pilosa* plant produces 40.05 ± 4.52 infructescences with each bearing 43.25 ± 1.23 fruits. Central achenes are dark brown, linear with four stiff awns, while peripheral achenes are black and shorter than central achenes. The number (*t* = 15.875, *P* < 0.001), mass (*t* = 7.702, *P* < 0.001) and length (*t* = 7.805, *P* < 0.001) of central achenes were significantly larger than peripheral achenes (Table 1), while the width (*t* = 8.016, *P* = 0.739) and height (*t* = 1.169, *P* = 0.709) were not significantly different. The ratio of central:peripheral achenes per infructescences was 8.67 ± 1.95.

**Table 1.** Comparison of the colour, number, mass and length of freshly matured central and peripheral achenes of *Bidens pilosa*. Measurements are mean ± SE. Different lowercase letters indicate significant differences between the two achene morphs by an independent sample *t*-test.

Morph	Colour	Number per infructescences	Seed mass (mg)	Seed length (cm)	Seed width (mm)	Seed height (mm)
Central	Dark brown	36.15 ± 1.49^a^	7.01 ± 026^a^	1.71 ± 0.06^a^	1.29 ± 0.03^a^	0.89 ± 0.03^a^
Peripheral	Black	7.2 ± 1.09^b^	4.60 ± 0.18^b^	1.23 ± 0.04^b^	1.15 ± 003^a^	0.84 ± 0.03^a^

### Imbibition of water by dimorphic achenes

**Figure 1.**
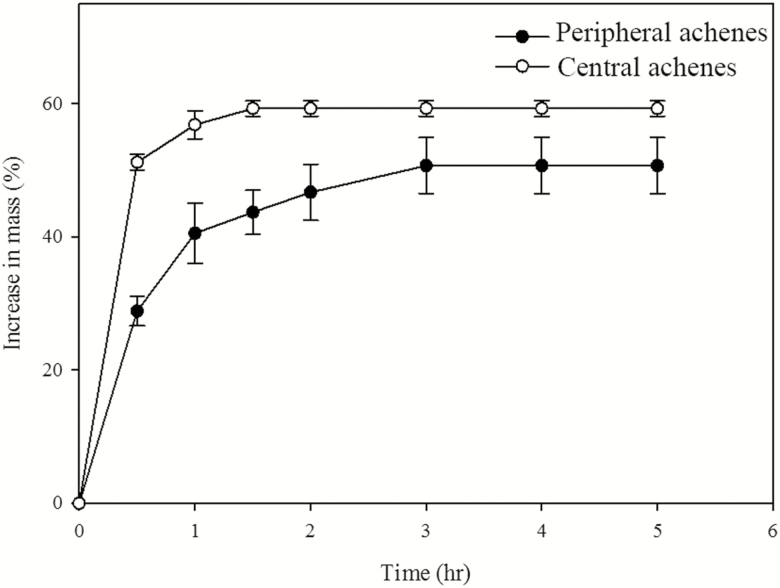
Imbibition curves for peripheral and central achenes of *Bidens pilosa.* Error bars are ±SE.

Central and peripheral achenes had different imbibition characters (*t* = 264.400, *P* < 0.001) (Fig. 1). Central achenes imbibed water quickly, and achene mass increased by 51.21 ± 1.64 % in 0.5 h and by 59.28 ± 1.16 % in 1.5 h, at which time water imbibition had reached its maximum. By contrast, peripheral achenes imbibed water slowly, with achene mass increased by 28.86 ± 2.23 in 0.5 h, 43.71 ± 3.34 in 1.5 h and 50.71 ± 4.20 in 3 h, by which time water uptake had peaked.

### Effects of light and temperature on germination of dimorphic achenes

**Figure 2.**
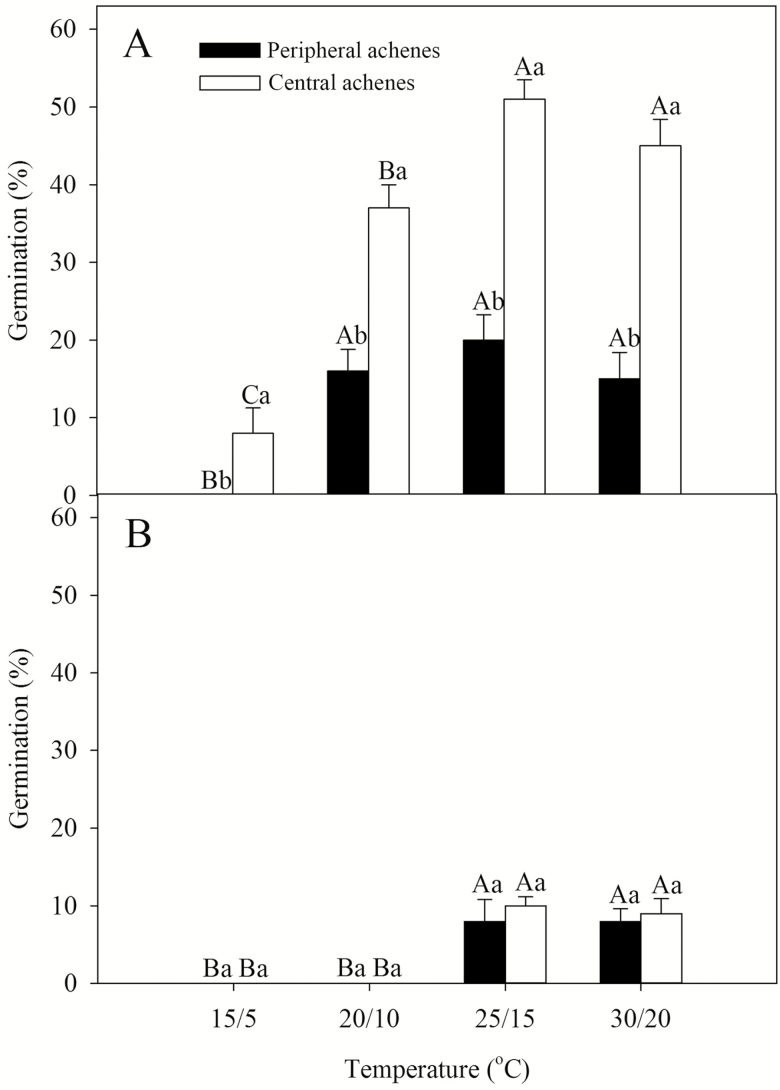
Effect of light and temperature on germination percentage (mean ± SE) of freshly matured dimorphic achenes of *Bidens pilosa*. (A) Germinated in light; (B) germinated in dark. The letters indicating significance are based on Tukey’s HSD after ANOVA. For each light treatment, different uppercase and lowercase letters indicate significant differences between treatments at different temperatures within each achene morph and between shout and central achenes within each temperature, respectively (5 % level).

A three-way ANOVA indicated that germination percentages were significantly affected by achene type, light conditions and temperature (Table 2). Germination of freshly matured central and peripheral achenes was ≤51.00 ± 2.52 % in light and in dark in all four temperature regimes. Both central and peripheral achenes had higher germination percentages in light condition than dark (Fig. 2). Central achenes had higher germination percentages than peripheral achenes in light in all temperature regimes, whereas no significant difference was observed in seeds germinated in the dark.

**Table 2.** Three-way ANOVA of effects of seed type, temperature, light condition and their interactions on seed germination of *Bidens pilosa*. MS, mean square; SS, sum of squares.

Source	d.f.	SS	MS	*F*	*P*
Light (L)	1	5402.250	5402.250	258.275	*P* < 0.001
Achene type (AT)	1	1722.250	1722.250	82.339	*P* < 0.001
Temperature (T)	3	3510.750	1170.250	55.948	*P* < 0.001
L × AT	1	1482.250	1482.250	70.865	*P* < 0.001
L × T	3	1368.750	456.250	21.813	*P* < 0.001
AT × T	3	312.750	104.250	4.984	0.004
L × AT × T	3	224.750	74.917	3.582	0.020

### Effects of cold stratification on germination of dimorphic achenes

**Figure 3.**
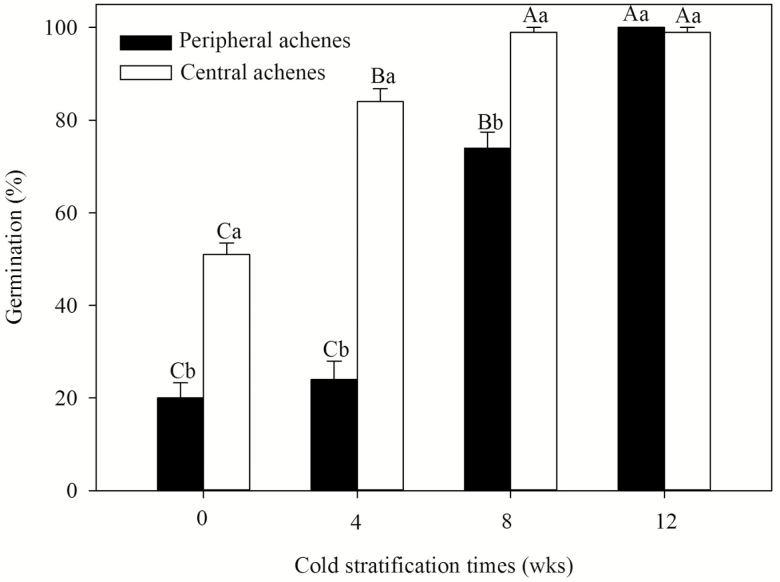
Germination percentages (mean ± SE) of central (white bars) and peripheral (black bars) achenes of *Bidens pilosa* at 25/15 °C after different weeks of cold stratification. The letters indicating significance are based on Tukey’s HSD after ANOVA. Different uppercase letters indicate significant differences among different cold stratification time and different lowercase letters indicate significant differences between central and peripheral achenes within each cold stratification time (5 % level).

Cold stratification significantly increased the germination percentages of dimorphic achenes; central achenes required 4 weeks of cold stratification to germinate to a percentage of >80 %, while peripheral achenes required 12 weeks (Fig. 3). A two-way ANOVA showed that germination percentage was significantly affected by achene type (*F* = 246.429, *P* < 0.001), cold stratification time (*F* = 252.292, *P* < 0.001) and their interaction (*F* = 49.807, *P* < 0.001).

### Effects of dry storage (after-ripening) on germination of dimorphic achenes

**Figure 4.**
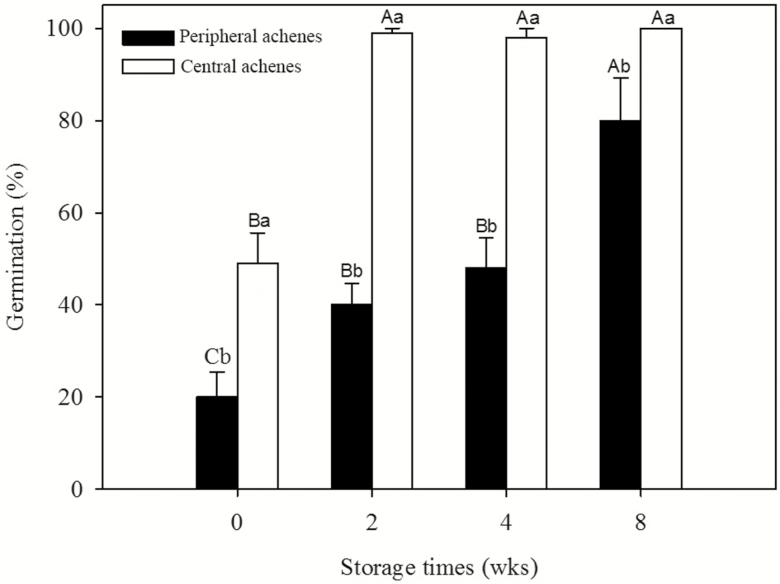
Effect of dry storage on germination percentage (mean ± SE) of freshly matured dimorphic achenes of *Bidens pilosa.* The letters indicating significance are based on Tukey’s HSD after ANOVA. Different uppercase and lowercase letters indicate significant differences between different dry storage time and central and peripheral achenes within each dry storage time, respectively (5 % level).

A two-way ANOVA showed that germination percentage was significantly affected by achene type (*F* = 109.812, *P* < 0.001), storage time (*F* = 38.175, *P* < 0.001) and their interaction (*F* = 5.753, *P* = 0.004). Two weeks of dry storage at room temperature increased germination of central achenes from 49 ± 6.61 to 99 ± 1 %. However, peripheral achenes stored for 0, 2, 4 and 8 weeks germinated to 20 ± 5.41, 41 ± 4.61, 48 ± 6.53 and 80 ± 3.27 %, respectively (Fig. 4).

### Effect of GA_3_ on germination of dimorphic achenes

**Figure 5.**
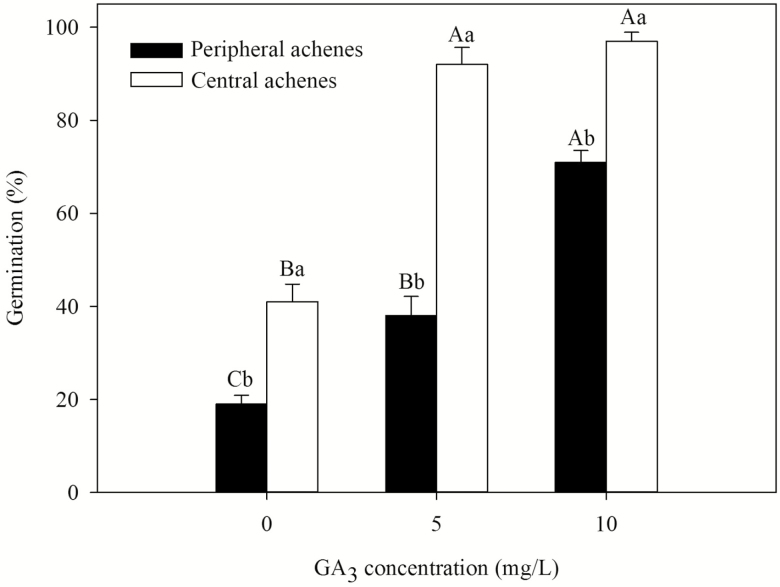
Effect of GA_3_ on germination percentage (mean ± SE) of freshly matured dimorphic achenes of *Bidens pilosa*. The letters indicating significance are based on Tukey’s HSD after ANOVA. Different and lower uppercase letters indicate significant differences between different GA_3_ concentrations and between central and peripheral achenes within each concentration, respectively (5 % level).

A two-way ANOVA showed that germination percentage was significantly affected by achene type (*F* = 177.341, *P* < 0.001), GA_3_ concentration (*F* = 153.477, *P* < 0.001) and their interaction (*F* = 15.545, *P* = 0.004). After 1 month of incubation, peripheral achenes incubated at 0 (control), 5 and 10 mg L^−1^ GA_3_ germinated to 19 ± 1.91, 38 ± 4.16 and 71 ± 2.51 %, respectively, and central achenes to 41 ± 3.78, 92 ± 3.65 and 97 ± 1.91 %, respectively (Fig. 5).

### Effect of osmotic potential on germination of dimorphic achenes

**Figure 6.**
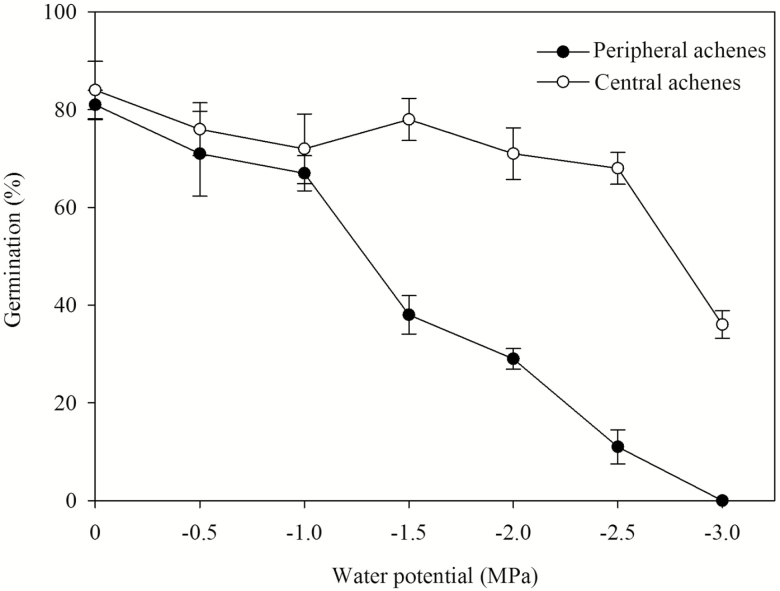
Effects of PEG treatments on germination percentage (mean ± SE) of freshly matured dimorphic achenes of *Bidens pilosa*.

A two-way ANOVA showed that germination after incubation for 30 days in various PEG solutions was significantly affected by achene type (*F* = 262.495, *P* < 0.001), PEG concentration (*F* = 47.790, *P* < 0.001) and their interaction (*F* = 18.206, *P* < 0.001). After 30 days of incubation, germination percentages of both peripheral and central achenes decreased with increasing osmotic potential. Germination of central achenes was higher than that of peripheral achenes in −1.5, −2.0, −2.5 and −3.0 MPa osmotic potentials. No peripheral achenes germinated in −3.0 MPa while those for central achenes were 36 ± 2.82 % (Fig. 6).

## Discussion

Many plant species inhabiting unpredictable environments have evolved a number of morphological and physiological adaptations to increase survival and fitness (Harper *et al.* 1970; Hughes 2018). *Bidens pilosa* is a serious weed species that often disturbed by human activity. The present study demonstrated that *B. pilosa* produced two distinct types of achenes, central and peripheral, which differ in morphology, germination behaviour, dormancy and osmotic tolerance. These differences represent the combination of different adaptive strategies in one plant and may have ecological significance for its successful survival.

Central achenes of *B. pilosa* have relatively long teeth and are exposed in the capitulum, whereas peripheral achenes have relatively short teeth and form against the involucral leaves. Moreover, central achenes were significantly heavier than peripheral achenes (Table 1). These variations in seed morphology usually result from the structure of the pericarp or the embryo/pericarp mass ratio (Weiss 1980; Forsyth and Brown 1982; Ellner and Shmida 1984; Venable and Levin 1985; Rocha 1996; Baskin and Baskin 2014) and often lead to variation in oxygen exchange, imbibition time and the loss of germination inhibitors, and may cause the different patterns of germination dynamics and patterns of soil seed bank (Baskin and Baskin 2014). A study of the masses and sizes/shapes (expressed as variance from a sphere) of diaspore of 97 species in the British in relation to longevity during burial showed that compact (low variance) dispersal units weighing <3 mg were persistent. Non-compact (high variance) dispersal units weighing >3 mg were short-lived (Thompson *et al.* 1993). Another study of 145 deciduous forest fragments in northwest Germany also indicated that seed mass decreased with number of seeds produced per plant. Further, seed longevity in the soil increased with a decrease in seed size (Kolb and Diekmann 2005). In *B. pilosa*, the loss of achene viability was faster for central than for peripheral achenes. Viability of freshly matured central and peripheral was 88 and 83 %, respectively. However, after 6 months of storage, viability of central and peripheral achenes decreased to 54 and 72 %, respectively (Rocha 1996). In mountain of Argentina, *B. pilosa* has two peaks of flowering and produces two different seed types with different mass and germination characteristics, the heavier seeds germinate faster than smaller seeds (Gurvich *et al.* 2004).

Dimorphic achenes of members of the genus *Bidens* have been reported to differ in dormancy and germination requirements in *B. bipinnata* (Dakshini and Aggarwal 1974), *B. gardneri* (Felippe 1990) and *B. odorata* (Corkidi *et al.* 1991). Since germination percentage of freshly matured peripheral and central achenes of *B. pilosa* incubated under four temperature regimes in light and in dark was ≤51.00 ± 2.52 % (Fig. 2), we can conclude that both achene morphs are dormant. Both freshly matured peripheral and central achenes are highly permeable to water and have a fully developed embryo. Although the amount and rate of water imbibition by peripheral achenes is lower than that of the central achenes, they are water permeable. The difference in the amount and the rate of water imbibition might result from the different sizes and morphology of the dimorphic achenes (Baskin and Baskin 2014). Furthermore, promotion of germination by cold stratification, GA_3_ and after-ripening in dry storage also indicates that these achenes have non-deep PD (*sensu*Baskin and Baskin 2014). These findings suggest a large percentage of freshly matured dormant achenes have a low growth potential in the embryo and cannot break the mechanical resistance of the achene coat. As a result, dry storage, GA_3_ and cold stratification treatments allowed the embryo to overcome the resistance of the seed coat, allowing the seed to germinate and the radicle to protrude (Cristaudo *et al.* 2007; Wang *et al.* 2008).

Seeds of many species with non-deep PD go through a series of changes in their capacities for physiological responses to various factors between dormancy and non-dormancy (Baskin and Baskin 2014; Soltani *et al.* 2017). During the time when seeds are released from dormancy, they initially enter a state of conditional dormancy (CD), where they will only germinate over a narrow range of conditions. As seeds progress from CD into being non-dormant, this range widens until finally the seeds will germinate over a full range of conditions (Baskin and Baskin 2014). Six types of non-deep PD are recognized by Soltani *et al.* (2017), based on the patterns of change in physiological responses to temperature during dormancy breaking. However, in the present study, no experiments were carried out on CD and the types of non-deep PD. Nevertheless, we could prove that after cold stratification during winter, achenes of *B. pilosa* gained the ability to germinate at 25/15 °C by late April and May. The high germination percentage after cold stratification and after-ripening might represent a transitional physiological state undergone between the states of primary dormancy and non-dormancy. Therefore, additional studies will be needed to quantify the effects of cold stratification and after-ripening on the germination of *B. pilosa* under more temperature regimes.

Both freshly matured central and peripheral achenes of *B. pilosa* had higher germination percentage in light conditions than dark, suggesting that light promoted germination and *B. pilosa* could germinated to higher percentages on the soil surface. Forsyth and Brown (1982) found that 100 % of central achenes germinated after 10 min of white light treatment or in a dark control; however, only 60 and 35 % of periphery achenes germinated after 10 min of white light treatment or in dark control, respectively. The germination percentage was higher than that in our study, perhaps because the seeds Forsyth and Brown (1982) used had been stored for 14 days in the dark after harvest and prior to the germination. The non-deep PD was partially broken by after-ripening. Similar results for the seeds of *B. bipinnata* were found by Brown and Mitchell (1984). Light promotes seed germination of many weed species (Woolley and Stoller 1978; Boyd and Van Acker 2004; Chauhan and Johnson 2008). Schutz *et al.* (2002) noted that light can act as an indicator of soil depth for seeds, allowing seeds near the surface to have higher germination percentages than deeply buried seeds. Cousens *et al.* (1993) had previously suggested that in no-till systems the ability of weedy species to germinate near the surface when stimulated by light has the potential to allow them to become problematic weeds; this occurs because most weed seeds stay near the soil surface in no-till systems after crops are planted. Central achenes of *B. pilosa* had higher germination percentages than peripheral achenes at 25/15 °C after 4 and 8 weeks of cold stratification (Fig. 3). That indicate peripheral achenes have a deeper dormancy than central achenes. In many heteromorphic species of Asteraceae, central achenes are less dormant than peripheral achenes (Venable 1995; Baskin and Baskin 2014). However, in seeds of *Emilia sonchifolia* (Marks and Akosim 1984) and *Leontodon saxatilis* (Brandel 2007), peripheral achenes are less dormant than central achenes. In *Synedrella nodiflora*, central achenes germinated to higher percentage than peripheral achenes in 26 °C and a 12-h daily photoperiod (Manilal and Unni 1978). However, at 27, 30 and 35 °C in continuous light and at 27 and 30 °C in darkness, peripheral achenes germinated to higher percentages than central achenes (Marks and Akosim 1984).

Differences in seed dormancy/germination may contribute to seedling recruitment by allowing plants to emerge in a broad range of regeneration niches (Grubb 1977; Coomes and Grubb 2003; Liyanage *et al.* 2016). In *B. pilosa*, freshly matured central achenes have a relatively low level of dormancy and may germinate across a wide range of temperatures similar to temperature of their natural habitat (Fig. 2). Therefore, Gutterman (2002) noted that central achenes of Asteraceae are expected to have an opportunistic germination strategy and peripheral achenes require an extended period of time to break dormancy. Peripheral achenes should have a conservative germination strategy that prevents all seeds from germinating simultaneously (Gutterman 2002). This allows an extended germination period and can produce a persistent seed bank and provide for the recruitment of seedlings over an extended period of time (Mandák and Pyšek 2001; Imbert 2002).

Polyethylene glycol solutions have been used to create water stresses similar to those which seeds receive in the field. In general, water potential of soils at the permanent wilting percentage is about −1.5 MPa (Baskin and Baskin 2014). However, sensitivity to water stress varies with the species. In distilled water, seeds of *Agriophyllum squarrosum*, *Aristida adscensionis*, *Artemisia ordosica*, *Bassia dasyphylla* and *Hedysarum scoparium* germinated to 99, 90, 100, 89 and 100 % in distilled water, respectively; in −5.0 MPa NaCl to 97, 55, 30, 90 and 0 %, respectively; and in −5.0 MPa PEG to 94, 82, 97, 96 and 79 %, respectively (Tobe *et al.* 1999). Germination of *B. pilosa* decreased with increasing osmotic potential of PEG, while central achenes germinated to a higher percentage than peripheral achenes at osmotic potentials lower than −1.0 MPa. More than 60 % central achenes could germinate in −2.5 MPa, but this fell to <20 % for peripheral achenes. This indicated peripheral achenes are far more sensitive to osmotic potential than central achenes and the central achenes may aid in population establishment in habitats with extreme drought stress.

Any morphological variation contributes to variation in dispersal distance (Greene and Johnson 1989) and any variation in dormancy contributes to variation in germination time (Baskin and Baskin 2014). Rocha (1996) demonstrated that central achenes of *B. pilosa* were more likely to attach to potential dispersers than peripheral ones. He found 40.4 % of the capitulum removed from the central positions; however, only 6.4 % of the capitulum had achenes removed from the peripheral positions (Rocha 1996). Therefore, peripheral achenes should disperse relatively poorly and so they can function to maintain a population within a favourable environment and allow for germination and growth (Rocha 1996). The central achenes act as colonizers, because they are better adapted to dispersal. Consequently, central achenes have a relatively high probability of dispersal into unfavourable habitats than peripheral achenes. Having a relatively high proportion of central achenes that remain dormant could provide a mechanism that ensures that the dispersed achenes fail to germinate simultaneously (Table 1). Some of these achenes will join the seed bank in a new habitat, or may be subjected to secondary dispersal (a similar process occurs in heterocarpic *Atriplex* species; Mandák 2003).

Heteromorphism that results in differences in dispersal, germination and post-germination behaviour of any two morphs is viewed as an adaptation (via a bet-hedging strategy) to an unpredictable environment (Venable 1985). In most species with dimorphic fruit/seed, each diaspore has strong dispersal ability and little (or no) seed dormancy (i.e. high risk, HR) and the other has a low (or no) dispersal ability along with a high (or relatively high) seed dormancy (i.e. low risk, LR); as a result, a high risk-low risk (HRLR) strategy often exists for seed dispersal/dormancy-germination (Venable and Lawlor 1980; Venable 1985; Baskin *et al.* 2013, 2014; Zhang *et al.* 2015). This allows a single genotype to maximize fitness via multiple phenotypes when the environments that it experiences are heterogeneous in time or space (Venable 1985; Schreiner 1993). For *B. pilosa*, trampling will affect its growth, leading to uncertainty in habitat stability. The central achenes are dispersed for longer distances and have relatively shallow seed dormancy, whereas peripheral achenes have low dispersal ability and relatively strong seed dormancy. The central morph will function by colonizing new sites at a distance from the mother plant as rapidly as possible, while seedlings from the peripheral morph stay near the mother plant, and will wait (by seed dormancy and delayed germination) for the best time to germinate. Thus, the central achenes are HR and the peripheral achenes are LR. Achene heteromorphism of *B. pilosa* allows the plants to adapt and reproduce with temporal and spatial variations in the surrounding environment, allowing this taxon to inhabit unpredictable habitats successfully.

## Conclusion

The present study determined that both central and peripheral achene of *B. Pilosa* exhibit non-deep PD that can be broken by cold stratification, GA_3_ and after-ripening in dry storage. Central achenes germinate more readily and had significantly higher germination percentages than the peripheral achenes, and may represent a HR strategy to ensure population establishment in heterogeneous habitats. Peripheral achenes of *B. Pilosa* germinate to a low percentage, and may represent a LR strategy to prevent all achenes from germinating simultaneously. In addition, central achenes were reported to have a higher dispersal ability than peripheral achenes (Rocha 1996). These differences within one capitulum may allow *B. pilosa* plants to limit the risk of mortality for its progeny, because this will increase the chance that at least some seedlings will germinate in a suitable place and time. Our results add to our understanding of the timing involved in weed seed germination in agricultural lands, as a part of weed management strategies.

## Sources of Funding

Funds for this study were provided by the National Natural Science Foundation of China (31800340); the Jiangsu Key Laboratory for Horticultural Crop Genetic Improvement of the P. R. China (2017023); and the Priority Academic Program Development from the Jiangsu Government.

## Contributions by the Authors

L.Y., K.Z. and J.T. conceived and designed the study; L.Y. and Y.Z. performed most of the experiments; K.Z. and Y.Z. analysed the data; Y.Z., K.Z. and J.T. wrote the manuscript.

## Conflict of Interest

None declared.

## Supporting Information

The original data information of figures and tables is available online (https://osf.io/q5bep/).

## References

[CIT0001] Baskin CC, Baskin JM 2014 Seeds: ecology, biogeography, and evolution of dormancy and germination, 2nd edn. San Diego, CA: Elsevier/Academic Press.

[CIT0002] Baskin JM, Lu JJ, Baskin CC, Tan DY 2013 The necessity for testing germination of fresh seeds in studies on diaspore heteromorphism as a life history strategy. Seed Science Research 23:83–88.

[CIT0003] Baskin JM, Lu JJ, Baskin CC, Tan DY, Wang L 2014 Diaspore dispersal ability and degree of dormancy in heteromorphic species of cold deserts of northwest China: a review. Perspectives in Plant Ecology, Evolution and Systematics 16:93–99.

[CIT0004] Boyd NS, Van Acker RC 2004 Seed germination of common weed species as affected by oxygen concentration, light, and osmotic potential. Weed Science 52:589–596.

[CIT0005] Brandel M 2007 Ecology of achene dimorphism in *Leontodon saxatilis*. Annals of Botany 100:1189–1197. 17901060 10.1093/aob/mcm214 PMC2759255

[CIT0006] Brown NAC, Mitchell JJ 1984 Germination of the polymorphic fruits of *Bidens bipinnata*. South African Journal of Botany 3:55–58.

[CIT0007] Chauhan BS, Johnson DE 2008 Germination ecology of southern crabgrass (*Digitaria ciliaris*) and India crabgrass (*Digitaria longiflora*): two important weeds of rice in tropics. Weed Science 56:722–728.

[CIT0008] Cheplick GP, Quinn JA 1988 Subterranean seed production and population responses to fire in *Amphicarpum purshii* (Gramineae). Journal of Ecology 76:263–273.

[CIT0009] Coomes DA, Grubb PJ 2003 Colonization, tolerance, competition and seed-size variation within functional groups. Trends in Ecology and Evolution 18:283–291.

[CIT0010] Corkidi L, Rincon E, Vazquez-Yanes C 1991 Effects of light and temperature on germination of heteromorphic achenes of *Bidens odorata* (Asteraceae). Canadian Journal of Botany 69:574–579.

[CIT0011] Cousens RD, Baweja R, Vaths J, Schofield M 1993 Comparative biology of cruciferous weeds: a preliminary study. In: Proceedings of the 10th Australian and 14th Asian-Pacific Weed Conference Brisbane, Australia: Weed Society of Queensland, 376–380.

[CIT0012] Cristaudo A, Gresta F, Luciani F, Restuccia A 2007 Effects of after-harvest period and environmental factors on seed dormancy of *Amaranthus* species. Weed Research 47:327–334.

[CIT0013] Dakshini KMM, Aggarwal SK 1974 Intracapitular cypsele dimorphism and dormancy in *Bidens bipinnata*. Biologia Plantarum 16:469–471.

[CIT0014] Ellner S, Shmida A 1984 Seed dispersal in relation to habitat in the genus *Picris* (Compositae) in Mediterranean and arid regions. Israel Journal of Botany 33:25–39.

[CIT0015] Felippe GM 1990 Germinaçao de *Bidens gardeneri* Baker, uma planta anual dos cerrados. Hoehnea 17:7–11.

[CIT0016] Forsyth C, Brown NAC 1982 Germination of the dimorphic fruits of *Bidens pilosa* L. New Phytologist 90:151–164.

[CIT0017] Greene DF, Johnson EA 1989 A model of wind dispersal of winged or plumed seeds. Ecology 70:339–347.

[CIT0018] Grubb PJ 1977 The maintenance of species-richness in plant communities: the importance of the regeneration niche. Biological Reviews 52: 107–145.

[CIT0019] Gurvich DE, Enrico L, Funes G, Zak MR 2004 Seed mass, seed production, germination and seedling traits in two phenological types of *Bidens pilosa* (Asteraceae). Australian Journal of Botany 52:647–652.

[CIT0020] Gutterman Y 2002 Survival strategies of annual desert plants. Berlin: Springer-Verlag.

[CIT0021] Harper JL, Lovell DH, Moore KG 1970 The shapes and sizes of seeds. Annual Review of Ecology and Systematics 1:327–351.

[CIT0022] Holm LG, Plucknett DL, Pancho JV, Herberger JP 1977 The world’s worst weeds: distribution and biology. Honolulu, HI: University Press Hawaii.

[CIT0023] Hughes PW 2018 Minimal-risk seed heteromorphism: proportions of seed morphs for optimal risk-averse heteromorphic strategies. Frontiers in Plant Science 9:1412. 30327659 10.3389/fpls.2018.01412 PMC6174283

[CIT0024] Imbert E 2002 Ecological consequences and ontogeny of seed heteromorphism. Perspectives in Plant Ecology, Evolution and Systematics 5:13–36.

[CIT0025] Kolb A, Diekmann M 2005 Effects of life-history traits on responses of plant species to forest fragmentation. Conservation Biology 19:929–938.

[CIT0026] Liu R, Wang L, Tanveer M, Song J 2018 Seed heteromorphism: an important adaptation of halophytes for habitat heterogeneity. Frontiers in Plant Science 9:1515. 30386364 10.3389/fpls.2018.01515 PMC6199896

[CIT0027] Liyanage GS, Ayre DJ, Ooi MK 2016 Seedling performance covaries with dormancy thresholds: maintaining cryptic seed heteromorphism in a fire-prone system. Ecology 97:3009–3018. 27870036 10.1002/ecy.1567

[CIT0028] Mandák B 2003 Germination requirements of invasive and non-invasive *Atriplex* species: a comparative study. Flora 198:45–54.

[CIT0029] Mandák B, Pyšek P 2001 Fruit dispersal and seed banks in *Atriplex sagittata*: the role of heterocarpy. Journal of Ecology 89:159–165.

[CIT0030] Manilal KS, Unni PN 1978 Performance variation with relation to morphological evolution in the dimorphic seeds of *Synedrella nodiflora* Gaertn. In: Sen DN, Bansal RP, eds. Environmental physiology and ecology of plants. Dehra Dun, India: B. Singh and M. P. Singh Publishers, 229–234.

[CIT0031] Marks MK, Akosim C 1984 Achene dimorphism and germination in three composite weeds. Tropical Agriculture 61:69–73.

[CIT0032] Rocha OJ 1996 The effects of achene heteromorphism on dispersal capacity of *Bidens pilosa* L. International Journal of Plant Science 157:316–322.

[CIT0033] Schreiner SM 1993 Genetics and evolution of phenotypic plasticity. Annual Review of Ecology and Systematics 24:35–68.

[CIT0034] Schutz W, Milberg P, Lamont BB 2002 Seed dormancy, after-ripening and light requirements of four annual Asteraceae in south-western Australia. Annals of Botany 90:707–714. 12451026 10.1093/aob/mcf250 PMC4240361

[CIT0035] Shi Z, Chen Y, Chen Y, Lin Y, Liu S, Ge X, Gao T, Zhu S, Liu Y, Humphries CJ, Yang Q, von Raab-Straube E, Gilbert MG, Nordenstam B, Kilian N, Brouillet L, Illarionova ID, Hind DJN, Jeffrey C, Bayer RJ, Kirschner J, Greuter W, Anderberg AA, Semple JC, Štěpánek J, Freire SE, Martins L, Koyama H, Kawahara T, Vincent L, Sukhorukov AP, Mavrodiev EV, Gottschlich G 2011 *Asteraceae* (Compositae). In: Wu ZY, Raven PH, Hong DY, eds. Flora of China, Volume 20–21 (Asteraceae). Beijing: Science Press.

[CIT0036] Soltani E, Baskin CC, Baskin JM 2017 A graphical method for identifying the six types of non-deep physiological dormancy in seeds. Plant Biology 19:673–682. 28612366 10.1111/plb.12590

[CIT0037] Thompson K, Band SR, Hodgson JG 1993 Seed size and shape predict persistence in soil. Functional Ecology 7:236–241.

[CIT0038] Tobe K, Zhang L, Omasa K 1999 Effects of NaCl on seed germination of five nonhalophytic species from a Chinese desert environment. Seed Science and Technology 27:851–863.

[CIT0039] Venable DL 1985 The evolutionary ecology of seed heteromorphism. American Naturalist 126:577–595.

[CIT0040] Venable DL 1995 Ecology of achene dimorphism in *Heterotheca latifolia*. III. Consequences of varied water availability. Journal of Ecology 73:757–763.

[CIT0041] Venable DL, Lawlor L 1980 Delayed germination and dispersal in desert annuals: escape in space and time. Oecologia 46:272–282. 28309684 10.1007/BF00540137

[CIT0042] Venable DL, Levin DA 1985 Ecology of achene dimorphism in *Heterotheca latifolia*. II. Demographic variation within populations. Journal of Ecology 73:743–755.

[CIT0043] Wang JG 2005 Shandong climate. Beijing: China Meteorological Press.

[CIT0044] Wang L, Huang Z, Baskin CC, Baskin JM, Dong M 2008 Germination of dimorphic seeds of the desert annual halophyte *Suaeda aralocaspica* (Chenopodiaceae), a C4 plant without Kranz anatomy. Annals of Botany 102:757–769. 18772148 10.1093/aob/mcn158 PMC2712381

[CIT0045] Weiss PW 1980 Germination, reproduction and interference in the amphicarpic annual *Emex spinosa* (L.) Campd. Oecologia 45:244–251. 28309535 10.1007/BF00346465

[CIT0046] Woolley JT, Stoller EW 1978 Light penetration and light-induced seed germination in soil. Plant Physiology 61:597–600. 16660344 10.1104/pp.61.4.597 PMC1091925

[CIT0047] Zhang K, Baskin JM, Baskin CC, Yang X, Huang Z 2015 Lack of divergence in seed ecology of two *Amphicarpaea* (Fabaceae) species disjunct between eastern Asia and eastern North America. American Journal of Botany 102:860–869. 26101412 10.3732/ajb.1500069

[CIT0048] Zhang K, Baskin JM, Baskin CC, Yang X, Huang Z 2017 Effect of seed morph and light level on growth and reproduction of the amphicarpic plant *Amphicarpaea edgeworthii* (Fabaceae). Scientific Reports 7:39886. 28071671 10.1038/srep39886 PMC5223113

